# Supplementary material for: Kidney stone disease increases the risk of cardiovascular events
Source: PLoS One. 2025 Sep 9;20(9):e0330069. doi: 10.1371/journal.pone.0330069 (PMC12419663; doi:10.1371/journal.pone.0330069)
Supplement: S1 Table — (DOCX) [file pone.0330069.s001.docx]

**S1 Table. Genetic instruments used in this Mendelian randomization study**

| **SNP** | **Exposure** | | |
| --- | --- | --- | --- |
|  | **Beta** | **SE** | **P value** |
| rs6667242 | 0.1976 | 0.0310 | 1.94E-10 |
| rs2924808 | 0.1275 | 0.0212 | 1.95E-09 |
| rs146948889 | -0.2410 | 0.0407 | 3.17E-09 |
| rs71606723 | 0.1220 | 0.0217 | 1.95E-08 |
| rs10051765 | 0.1432 | 0.0208 | 6.10E-12 |
| rs1010269 | 0.1556 | 0.0268 | 6.67E-09 |
| rs2585442 | 0.1391 | 0.0220 | 2.47E-10 |
| rs2776288 | -0.1270 | 0.0215 | 3.46E-09 |
| rs9608071 | 0.1500 | 0.0256 | 4.33E-09 |

SNP, single nucleotide polymorphism; SE, standard error
