# Supplementary figures and images for: Kidney stone disease increases the risk of cardiovascular events
Source: PLoS One. 2025 Sep 9;20(9):e0330069. doi: 10.1371/journal.pone.0330069 (PMC12419663; doi:10.1371/journal.pone.0330069)

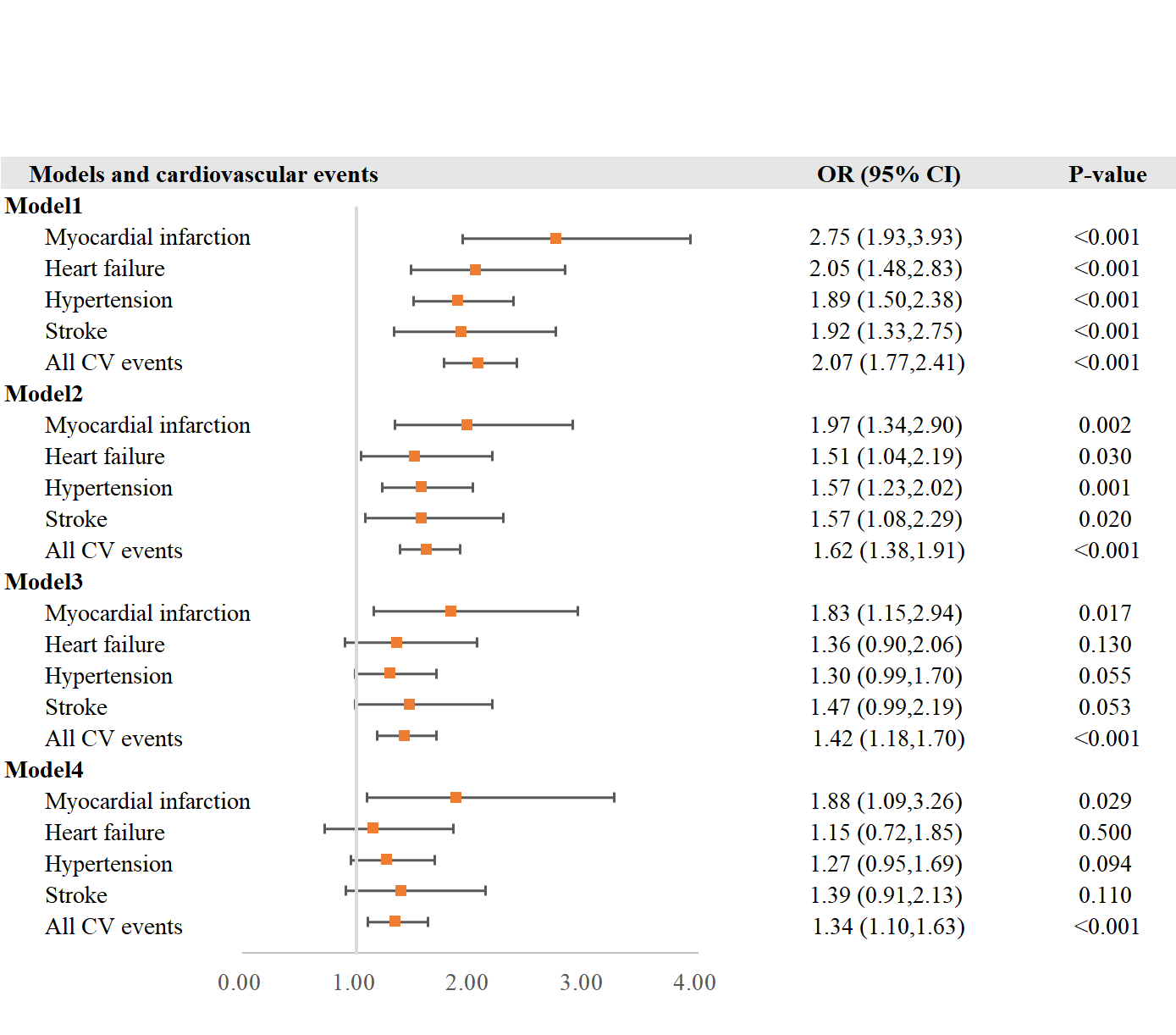

Supplement: S1 Fig — Model 1 adjusted for none. Model 2 adjusted for age, gender, race and education attainment. Model 3 further adjusted for BMI category, smoked 100 cigarettes, total calories, calcium intake, CRP status, eGFR, diabetes, gout and hypercholesterolemia. Model 4 further adjusted for other CVDs beyond the primary outcome. Abbreviations: OR, Odds Ratio; CI, confidence interval; CVDs, cardiovascular diseases. (TIF) [file pone.0330069.s002.tif]

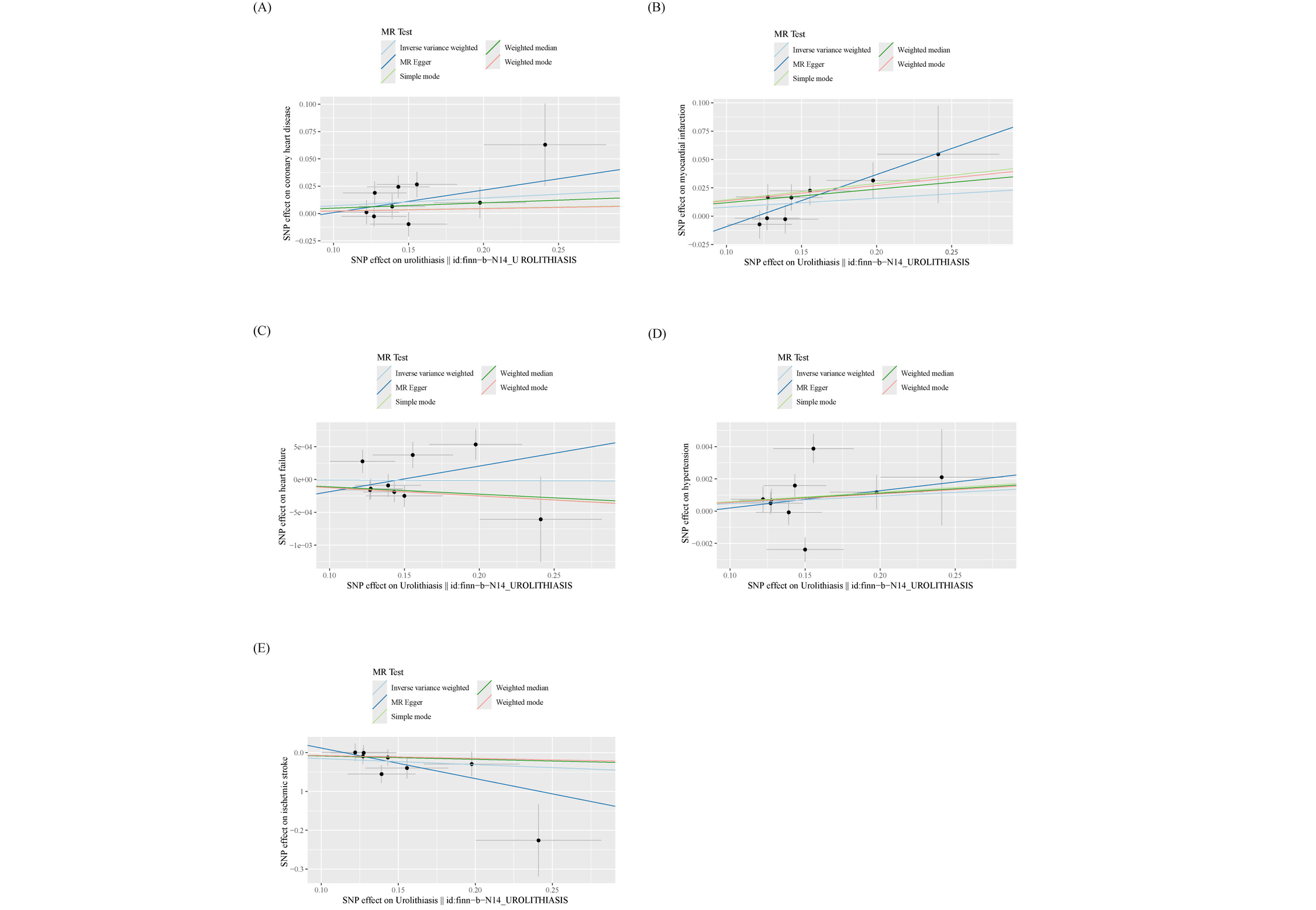

Supplement: S2 Fig — (A) Coronary heart disease. (B) Myocardial infarction. (C) Heart failure. (D) Hypertension. (E) Stroke. (TIF) [file pone.0330069.s003.tif]

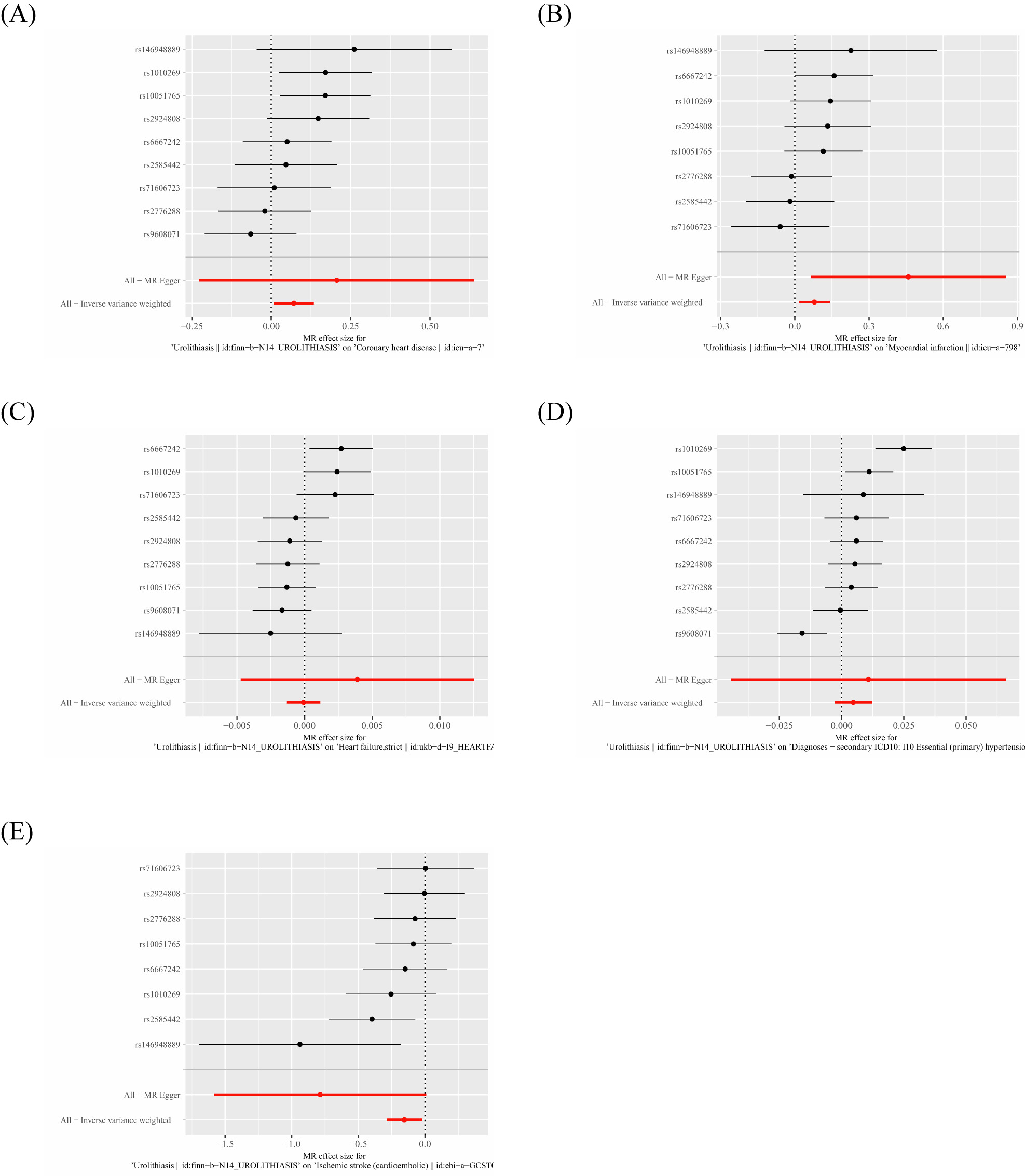

Supplement: S3 Fig — (A) Coronary heart disease. (B) Myocardial infarction. (C) Heart failure. (D) Hypertension. (E) Stroke. (TIF) [file pone.0330069.s004.tif]
