# Supplementary material for: Kidney stone disease increases the risk of cardiovascular events
Source: PLoS One. 2025 Sep 9;20(9):e0330069. doi: 10.1371/journal.pone.0330069 (PMC12419663; doi:10.1371/journal.pone.0330069)
Supplement: Attachment — Submitted filename: Response to Reviewers.docx [file pone.0330069.s006.docx]

Dear Editors and Reviewers:

Thank you for your letter and for the reviewers’ comments concerning our manuscript entitled “Kidney stone disease increases the risk of cardiovascular events” (ID: PONE-D-25-10263). Those comments are all valuable and very helpful for revising and improving our paper, as well as the important guiding significance to our researches. We have studied comments carefully and have made correction which we hope meet with approval.

To easily distinguish my answers from reviews’ comments, we highlight all of our answers in blue while keeping your letter and reviews’ comments in black in the Response letter.

Thank you again for your time and consideration.

Looking forward to hearing from you.

**Referee Comments to Author**

**Reviewer #1:**

**Comments to the Author**

1.Prevalence - provide a number in your introduction.

***Reply:*** We gratefully appreciate for your valuable suggestion.

According to your comments, we have added a detailed description of the prevalence of kidney stones in the introduction section, and the revised sentence is presented as follows: “The worldwide prevalence of nephrolithiasis is estimated to range between 7.2% and 7.7%[1]” (pages 1-2).

**2.** Diagnosis of kidney stone? was it made by Imaging or for someone who required intervention - lithotripsy? please clarify.

***Reply:*** We extend our sincere appreciation for the reviewer's insightful suggestions.

In the NHANES database, the diagnosis of kidney stones was ascertained using the Kidney Conditions-Urology survey within the questionnaire data. This survey was administered by trained interviewers in participants' homes, employing a computer-assisted personal interview (CAPI) system. Participants were queried with the question, "Have you ever had a kidney stone?" An affirmative response was used to classify the participant as having a history of kidney stones. We have added the relevant content to the methodology section of the manuscript.

In the context of Mendelian randomization, the cohort of individuals with urolithiasis was derived from the Finnish database, with genetic instrumental variables established based on samples exhibiting urolithiasis phenotypes. In the FinnGen consortium, 5347 cases of kidney stones were diagnosed based on N20 in the International Classification of Diseases Tenth Edition (ICD-10) and self-reported surgical codes. The accuracy of these codes for defining a kidney stone has been previously validated[2].

**3.** Biggest limitation is no known renal function - no creatinine values? which would be the biggest confounder. This is well established that renal stone will cause CKD and CKD is a major risk for cardiovascular disease. is it possible to obtain lab values?

***Reply:*** We express our gratitude for the reviewer's insightful suggestions.

In response, we have incorporated the creatinine values of the patients and employed the Chronic Kidney Disease Epidemiology Collaboration (CKD-EPI) equation to calculate the estimated glomerular filtration rate (eGFR) based on serum creatinine (Scr) levels. We excluded participants with baseline eGFR values below 15 ml/min/1.73 m², as well as those who were undergoing dialysis or had received a kidney transplant at baseline. For subsequent analyses, we included participants with eGFR values categorized as ≥60, 45-59.9, 30-44.9, and 15-29.9 ml/min/1.73m². After accounting for the confounding variable of renal creatinine levels, our analysis reveals a persistent significant association between kidney stone disease and myocardial infarction, as detailed in the results section.

**Reviewer #2:**

**Comments to the Author**

1.Given the topic of kidney stone data and evaluating associations, the data is presented in an extremely complex way. For an average reader of this article, it has dense amount of statistical analysis without clarity.

***Reply:*** **We express our gratitude for the insightful feedback offered by the reviewers. In response, we have conducted a thorough reanalysis of the manuscript's statistical components, ensuring that the presentation is as clear and comprehensible as possible. Additionally, we have incorporated several images and tables into the revised manuscript to enhance its clarity and accessibility.**

2. Table 1 also needs better explanation. Text states that 7377 individuals selected but N was 7282 (including the Forest plot).

***Reply:*** We gratefully appreciate for your valuable suggestion.

Firstly, the description of 7377 individuals in the text is incorrect and the original text should be revised to “In our observational research, we selected 7,282 individuals”. Table 1 illustrates the baseline characteristics of participants by kidney stone disease status. Following the re-conduct of the statistical analysis, the sample size (n) should be modified to 7210. We have conducted a reanalysis and detailed corrections in the manuscript.

3. Secondly, what is the "N" in No or Yes? That has no explanation and these multiple "N" categories are confusing.

***Reply:*** We extend our sincere appreciation for the reviewer's insightful suggestions.

The NHANES database employs a complex, multi-stage probability sampling design, resulting in unequal probabilities of individual selection. Consequently, it is necessary to adjust for these unequal probabilities in subsequent analyses to ensure that the individual random sampling aligns with the overall distribution of sample characteristics.

In Table 1 of the original text, the "N" associated with the "No" or "Yes" responses denote the weighted population size, which differs from the actual sample size, n, that is unweighted. Typically, N=weight * n, and the proportions derived from N more accurately reflect the distribution of the actual population in the United States. In Table 1, n represents the number of unweighted observed samples, while the percentage (%) reflects the weighted proportion. To mitigate potential confusion arising from varied terminologies, we have opted to eliminate the weighted sample size N and consistently represent the population size using the unweighted sample size n. But subsequent analysis is based on the situation that can reflect the actual population distribution.

4. In section 3.1.2 authors state heart failure was not significant but In Table 2, Heart Failure appears to be statistically significant. There seems to be discrepancy.

***Reply:*** Thank you for the valuable feedback provided by the reviewer. It may be that I did not clearly state the OR meaning of each variable.

Table 2 in the original manuscript is a multivariate model controlling for age, sex, race, education attainment, BMI category, CRP status, diabetes, hypertension, heart failure, gout, hypertension, hypercholesterolemia, smoked 100 cigarettes, total calories, calcium intake. Therefore, the meaning of heart failure OR here should be that patients with heart failure have a higher adjusted OR for AMI (OR, 9.15 [95% CI, 5.81-15.5]).

To address potential misunderstandings associated with the original Table 2, we have revised it and presented an updated version. In this new Table 2, we have adjusted four models. Model 1 includes no variable adjustments, whereas Model 2 accounts for demographic characteristics such as age, gender, race, and educational attainment. Model 3 incorporates additional adjustments for BMI category, history of smoking (defined as having smoked 100 cigarettes), CRP status, eGFR, diabetes, gout, hypercholesterolemia, total caloric intake and calcium intake. Furthermore, Model 4 includes adjustments for other cardiovascular diseases beyond the primary outcome. The results indicate that the association between kidney stones and myocardial infarction remains statistically significant across all four models. For a detailed result, please refer to Section 3.1.2 of the article, which addresses the relationship between kidney stone disease (KSD) and cardiovascular events.

**Reply Table 1 (Table 2 in the revised manuscript) The association between kidney stone disease and cardiovascular events.**

| **Models** | **Cardiovascular Events** | | | | |
| --- | --- | --- | --- | --- | --- |
|  | Myocardial infarction | Heart failure | Hypertension | Stroke | All CV events |
|  | OR (95%CI) | OR (95%CI) | OR (95%CI) | OR (95%CI) | OR (95%CI) |
| Model1 | 2.75 (1.93, 3.93) *** | 2.05 (1.48, 2.83) *** | 1.89 (1.50, 2.38) *** | 1.92 (1.33, 2.75) *** | 2.07(1.77,2.41) *** |
| Model2 | 1.97 (1.34, 2.90) ** | 1.51 (1.04, 2.19) * | 1.57 (1.23, 2.02) *** | 1.57 (1.08, 2.29) * | 1.62(1.38,1.91) *** |
| Model3 | 1.83 (1.15, 2.94) * | 1.36 (0.90, 2.06) | 1.30 (0.99, 1.70) | 1.47 (0.99, 2.19) | 1.42(1.18,1.70) *** |
| Model4 | 1.88 (1.09, 3.26) * | 1.15 (0.72,1.85) | 1.27 (0.95, 1.69) | 1.39 (0.91, 2.13) | 1.34(1.10,1.63) *** |

OR: odds ratio. 95% CI: 95% confidence interval.

* p < 0.05

** p < 0.01

*** p < 0.001

Model 1: crude model. Model 2: adjusted for demographic characteristics including age, gender, race and education attainment. Model 3: further adjusted for age, gender, race, education attainment, BMI category, CRP status, diabetes, gout, hypercholesterolemia, smoked 100 cigarettes, total calories, eGFR and calcium intake. Model 4: further adjusted for age, gender, race, education attainment, BMI category, CRP status, diabetes, gout, hypercholesterolemia, smoked 100 cigarettes, total calories, eGFR, calcium intake and other CVDs.

**Reviewer #3:**

**Comments to the Author**

**1.** The manuscript explores a relevant topic with valuable implications and is based on a sound methodological framework. However, improvements are needed in clarity, organization, data presentation, and language to strengthen its overall quality and impact.
***Reply:*** We express our sincere gratitude to the reviewers for their constructive feedback aimed at improving the clarity, organization, data presentation, and linguistic quality of our manuscript. These invaluable suggestions will undoubtedly enhance the rigor and accessibility of our research.

In terms of clarity, we have removed seemingly plausible conclusions, made further modifications to the results section, and redrawn unclear charts. For details, please refer to the results section of the manuscript.

In terms of organization, the introduction and discussion sections have been restructured, and the logic and causal inference sections have been further revised and integrated. For specific details, please consult the manuscript.

In terms of data presentation, we have added a process analysis on Mendelian randomization. This will facilitate a clearer comprehension of the Mendelian randomization process and its associated implications for readers (**Fig 1, Fig 2 in the revised manuscript)**. We have revised the forest plot illustrating the interactions within the subgroup analysis to enhance the clarity and immediate comprehensibility of the data (**Fig 2, S1 Fig in the revised manuscript)**. Besides, to enhance the rigor of the Mendelian randomization process, we have incorporated pertinent scatter plots and forest plots into the supplementary materials (**Fig 3, S2 Fig in the revised manuscript and Fig 4, S3 Fig in the revised manuscript))**.

In terms of language, we apologize for the poor language of our manuscript. We worked on the manuscript for a long time and the repeated addition and removal of sentences and sections obviously led to poor readability. We have now worked on both language and readability and have also involved native English speakers for language corrections. We really hope that the flow and language level have been substantially improved. we have polished our manuscript carefully and corrected the grammatical, styling, and typos found in our manuscript.

**Fig 1 (Fig 2 in the revised manuscript) The study design of two‐sample MR analysis.** Assumption 1: IVs are directly associated with urolithiasis. Assumption 2: IVs are dependent of any potential confounders that impact exposure and outcome. Assumption 3: SNPs must only affect cardiovascular diseases via urolithiasis.

**Fig 2 (S1 Fig in the revised manuscript) Multi -state results: ORs and 95% CIs of cardiovascular events.**

Model 1 adjusted for none. Model 2 adjusted for age, gender, race and education attainment. Model 3 further adjusted for BMI category, smoked 100 cigarettes, total calories, calcium intake, CRP status, eGFR, diabetes, gout and hypercholesterolemia. Model 4 further adjusted for other CVDs beyond the primary outcome.

Abbreviations: OR, Odds Ratio; CI, confidence interval; CVDs, cardiovascular diseases.

**Fig 3 (S2 Fig in the revised manuscript) Scatter plots for MR analyses of the causal effect of kidney stone disease on cardiovascular events.** (A) Coronary heart disease. (B) Myocardial infarction. (C) Heart failure. (D) Hypertension. (E) Stroke.

**Fig 4 (S3 Fig in the revised manuscript) Forest plot to visualize causal effect of each single SNP of kidney stone disease and cardiovascular events.** (A) Coronary heart disease. (B) Myocardial infarction. (C) Heart failure. (D) Hypertension. (E) Stroke.

**Major comments**

**1.** The objectives of the study need to be stated more clearly in the introduction. Currently, the aims are somewhat vague and scattered.

***Reply:*** We express our gratitude for the reviewer's insightful suggestions. In response, we have restructured the introduction and incorporated a distinct paragraph to elucidate the purpose of our research. The details are as follows: “… Studies have demonstrated that the development of nephrolithiasis is affected by genetic predispositions, environmental conditions, dietary habits, physical activity levels, and various other determinants. Kidney stones are linked to systemic conditions, including hypertension, diabetes, and dyslipidemia, which are recognized as risk factors for cardiovascular disease (CVD). Consequently, it is imperative to elucidate the relationship between kidney stones and cardiovascular disease. … Given the rising incidence of kidney stones over the years, a precise evaluation of their associated cardiovascular risks is essential for alerting patients and facilitating effective health interventions. Patients with kidney stones should focus on modifying cardiovascular risk factors to mitigate the comorbidity risk of both conditions. Furthermore, elucidating the relationship between KSD and cardiovascular events is of substantial importance for advancing our understanding of the etiology of these diseases. We conducted a cross-sectional study based on NHANES to determine the relationship between kidney stones and cardiovascular events. Following this, a two-sample Mendelian randomization (MR) was applied to further explore the causal relationship between these two conditions, employing genetic markers associated with kidney stones as instrumental variables to infer causal relationships with specific cardiovascular disease outcomes. This approach is more resistant to biases associated with confounding variables and the issue of reverse causality that can plague traditional observational research” (Page 9-10 in the revised manuscript).

2. More details should be provided regarding the study design and participant selection. For instance, information about sample size calculation, inclusion/exclusion criteria,

***Reply:*** *Thank you very much indeed for your comments.*

Owing to the cross-sectional design of the study and accompanying literature review, the global prevalence of kidney stones is estimated to be approximately 7.2-7.7%. The sample size for a cross-sectional study is calculated using the formula: n=(Zσ2×pq)/d2, where Zσ represents the significance test statistic. For a significance level α of 0.05, Zσ is 1.96. Here, p is the estimated prevalence of kidney stones, set at 8%, q=1-p, and d is the allowable error, specified as 0.02. Using these parameters, the minimum required sample size n is calculated to be 707. Accounting for a potential data and quality loss of 10%, the adjusted minimum sample size required is 785. Based on the established inclusion and exclusion criteria, a total of 7210 participants were included in the study, thereby exceeding the minimum sample size requirement. In my study, logistic regression analysis was used with 16 variables. According to previous research, the sample size should be 5, 10, or 20 times the number of variables[3]. A sample size of 320 would comply with the strictest empirical rule. The inclusion of 7210 research subjects also meets the sample size requirements for multivariate regression analysis.

Due to the lack of relevant content on sample size calculation in the methodology section of most literature, our description of sample size is as follows: “This study selected two NHANES cycles from 2007 to 2010 to assess the association between kidney stones and cardiovascular events. A total of 20,688 participants were initially enrolled. After excluding participants aged < 18 years (n = 7933), those with incomplete data on KSD (n = 645) or cardiovascular events (n = 61), those with incomplete data on other covariates (n = 4767), and those with recorded estimated glomerular filtration rate (eGFR) values under 15 ml/min/1.73 m², as well as those who had undergone dialysis or kidney transplantation (n = 72). 7210 participants were included in our final analysis”.

In a word, our inclusion criteria include: This study selected two NHANES cycles from 2007 to 2010 to assess the association between kidney stones and cardiovascular events. A total of 20688 participants were initially enrolled. Exclusion criteria include: (A) participants aged <18 years(n = 7933), (B) participants with incomplete data on kidney stone disease (n = 645) or cardiovascular events (n = 61), (C) participants with incomplete data on other covariates (n = 4767), and participants with recorded estimated glomerular filtration rate (eGFR) values under 15 ml/min/1.73 m², as well as those who had undergone dialysis or kidney transplantation (n = 72).

3. Tables and figures need to be formatted more clearly, with consistent use of labels, units, and statistical indicators (e.g., p-values, confidence intervals). Some of the figures are difficult to interpret due to lack of descriptive legends and clarity.

*Reply*: We agree with this comment and have updated Table 1. We have implemented the relevant modifications to the description in the revised manuscript: “In our observational study, we selected 7,210 individuals. Table 1 presents the baseline characteristics of the participants according to KSD status. We compared the demographic differences between participants with and without kidney stones (Table 1). Participants with kidney stones were significantly more likely to be older (55.7 versus 50.6 years), have a higher incidence of diabetes mellitus (17.5% versus 9.3%), a higher risk of gout (9.8% versus 4.1%), and a higher risk of hypercholesterolemia (51.3% versus 41.1%) (all P < 0.001).”

**Reply Table 2 (Table 1 in the revised manuscript) Baseline characteristics of participants categorized by kidney stone disease status in the NHANES 2007–2010 study.**

| **Variable** | **Overall** | **No Stone** | **Stone** | **P-value** |
| --- | --- | --- | --- | --- |
| Sex |  |  |  | <0.001 |
| Male | 3366 (45.5) | 2893 (44.0) | 473 (57.4) |  |
| Female | 3844 (54.5) | 3529 (56.0) | 315 (42.6) |  |
| Age | 51.2 (15.5) | 50.6 (15.6) | 55.7 (14.4) | <0.001 |
| Race |  |  |  | <0.001 |
| Mexican American | 1047 (5.9) | 960 (6.1) | 87 (4.2) |  |
| Other Hispanic | 722 (4.1) | 641 (4.0) | 81 (4.1) |  |
| Non-Hispanic White | 3807 (75.0) | 3301 (74.1) | 506 (82.4) |  |
| Non-Hispanic Black | 1336 (10.0) | 1245 (10.6) | 91 (5.4) |  |
| Others | 298 (5.0) | 275 (5.2) | 23 (3.9) |  |
| Education attainment |  |  |  | 0.017 |
| Less Than 9th Grade | 793 (5.0) | 695 (4.9) | 98 (6.3) |  |
| 9-11th Grade | 1042 (10.9) | 923 (10.8) | 119 (11.1) |  |
| High School Grad/GED | 1599 (22.0) | 1402 (21.6) | 197 (25.3) |  |
| Some College or AA degree | 2043 (30.6) | 1822 (30.4) | 221 (32.6) |  |
| College Graduate or above | 1733 (31.5) | 1580 (32.3) | 153 (24.7) |  |
| BMI category (%) |  |  |  | 0.011 |
| Underweight | 78 (1.2) | 74 (1.3) | 4 (0.9) |  |
| Normal weight | 1705 (26.1) | 1568 (27.0) | 137 (17.8) |  |
| Overweight | 2483 (34.2) | 2211 (34.0) | 272 (35.7) |  |
| Obesity | 2944 (38.5) | 2569 (37.7) | 375 (45.6) |  |
| CRP status |  |  |  | 0.6 |
| Low | 6463 (91.0) | 5759 (91.1) | 704 (90.5) |  |
| High | 747 (9.0) | 663 (8.9) | 84 (9.5) |  |
| Smoked 100 cigarettes |  |  |  | 0.14 |
| Yes | 3325 (45.0) | 2929 (44.6) | 396 (48.2) |  |
| No | 3885 (55.0) | 3493 (55.4) | 392 (51.8) |  |
| Intake |  |  |  |  |
| Total calories | 2056.2(803.0) | 2,047.5(798.4) | 2,128.8(837.2) | 0.2 |
| Calcium | 967.0(505.2) | 971.4(512.9) | 929.9(434.7) | 0.4 |
| Sodium | 3,455.1(1,514.8) | 3,439.6(1,492.5) | 3,584.4(1,684.7) | 0.2 |
| Total plain water drank | 993.9(937.3) | 1,001.1(936.8) | 933.7(940.2) | 0.047 |
| eGFR |  |  |  | 0.4 |
| 15-29.9 | 44 (0.4) | 39 (0.4) | 5 (0.7) |  |
| 30-44.9 | 154 (1.2) | 130 (1.2) | 24 (1.6) |  |
| 45-59.9 | 373 (3.0) | 319 (3.0) | 54 (3.5) |  |
| ≥60 | 6639 (95.2) | 5934 (95.4) | 705 (94.2) |  |
| Diabetes |  |  |  | <0.001 |
| Yes | 1081 (10.2) | 897 (9.3) | 184 (17.5) |  |
| No | 6129 (89.8) | 5525 (90.7) | 604 (82.5) |  |
| Myocardial infarction |  |  |  | <0.001 |
| Yes | 394 (3.9) | 310 (3.3) | 84 (8.7) |  |
| No | 6816 (96.1) | 6112 (96.7) | 704 (91.3) |  |
| Heart failure |  |  |  | <0.001 |
| Yes | 270 (2.6) | 222 (2.4) | 48 (4.7) |  |
| No | 6940 (97.4) | 6200 (97.6) | 740 (95.3) |  |
| Hypertension |  |  |  | <0.001 |
| Yes | 3149 (37.1) | 2709 (35.5) | 440 (51.0) |  |
| No | 4061 (62.9) | 3713 (64.5) | 348 (49.0) |  |
| Stroke |  |  |  | <0.001 |
| Yes | 334 (3.4) | 271 (3.1) | 63 (5.8) |  |
| No | 6876 (96.6) | 6151 (96.9) | 725 (94.2) |  |
| Gout |  |  |  | <0.001 |
| Yes | 420 (4.7) | 329 (4.1) | 91 (9.8) |  |
| No | 6790 (95.3) | 6093 (95.9) | 697 (90.2) |  |
| Hypercholesterolemia |  |  |  | 0.001 |
| Yes | 3229 (42.2) | 2810 (41.1) | 419 (51.3) |  |
| No | 3981 (57.8) | 3612 (58.9) | 369 (48.7) |  |

Table 2 will be revised and updated for presentation purposes, ensuring the use of consistent labels, units, and statistical indicators. Additionally, descriptive explanations for each model will be incorporated. The details are as follows: “We investigated the association between KSD and cardiovascular risks using logistic regression models. In model 1, the risk of cardiovascular events among patients with a kidney stone history was higher compared to those without a kidney stone history. Compared with non-kidney stone individuals, ORs (95% CI) for risks of MI, heart failure, hypertension, and stroke among patients with kidney stones were 2.75 [95% CI, 1.93–3.93], 2.05 [95% CI, 1.48–2.83], 1.89 [95% CI, 1.50–2.38], and 1.92 [95% CI, 1.33–2.75], respectively. In model 2, the association between kidney stones and cardiovascular events risk remained significant (Fig. 2). In models 3 and 4, kidney stone was only associated with an increased risk of AMI, but not heart failure, hypertension, or stroke. In model 4, significant associations were also observed between the development of kidney stones and the composite outcome of the individual events (OR, 1.34 [95% CI, 1.10–1.63]) .”

**Reply Table 1 (Table 2 in the revised manuscript) The association between kidney stone disease and cardiovascular events.**

| **Models** | **Cardiovascular Events** | | | | |
| --- | --- | --- | --- | --- | --- |
|  | Myocardial infarction | Heart failure | Hypertension | Stroke | All CV events |
|  | OR (95%CI) | OR (95%CI) | OR (95%CI) | OR (95%CI) | OR (95%CI) |
| Model1 | 2.75 (1.93, 3.93) *** | 2.05 (1.48, 2.83) *** | 1.89 (1.50, 2.38) *** | 1.92 (1.33, 2.75) *** | 2.07(1.77,2.41) *** |
| Model2 | 1.97 (1.34, 2.90) ** | 1.51 (1.04, 2.19) * | 1.57 (1.23, 2.02) *** | 1.57 (1.08, 2.29) * | 1.62(1.38,1.91) *** |
| Model3 | 1.83 (1.15, 2.94) * | 1.36 (0.90, 2.06) | 1.30 (0.99, 1.70) | 1.47 (0.99, 2.19) | 1.42(1.18,1.70) *** |
| Model4 | 1.88 (1.09, 3.26) * | 1.15 (0.72,1.85) | 1.27 (0.95, 1.69) | 1.39 (0.91, 2.13) | 1.34(1.10,1.63) *** |

OR: odds ratio. 95% CI: 95% confidence interval.

* p < 0.05

** p < 0.01

*** p < 0.001

Model 1: crude model. Model 2: adjusted for demographic characteristics including age, gender, race and education attainment. Model 3: further adjusted for age, gender, race, education attainment, BMI category, CRP status, diabetes, gout, hypercholesterolemia, smoked 100 cigarettes, total calories, eGFR and calcium intake. Model 4: further adjusted for age, gender, race, education attainment, BMI category, CRP status, diabetes, gout, hypercholesterolemia, smoked 100 cigarettes, total calories, eGFR, calcium intake and other CVDs.

4. The discussion would benefit from a deeper integration of the study’s findings with existing literature, as there is little critical comparison with prior research. The implications of the results and potential directions for future studies are also underdeveloped.

*Reply*: We express our gratitude for the reviewer's insightful suggestions. In response, we have restructured the discussion section to systematically compare Elmferdt's seminal study on the association between kidney stones and cardiovascular disease, three extensive prospective cohort studies conducted by Ferraro and Taylor, and a longitudinal study utilizing a population-based cohort database by Chien Yi Hsu. We have added the significance of the research and future research directions in the conclusion section, as detailed below: “In light of the observed correlation, it is advisable to implement screening protocols for cardiac dysfunction in patients with kidney stones. Patients diagnosed with urolithiasis may have an additional risk factor for CVD. This finding introduces novel perspectives for incorporating kidney stones into the risk stratification management of cardiovascular diseases. Evidence indicates that implementing lifestyle modifications may contribute to the prevention of both conditions[4]. By initiating early lifestyle interventions, such as DASH diet and exercise for weight reduction, alongside established risk factor prevention and control strategies[5], namely, the management of blood pressure, blood glucose, and lipid levels, this comprehensive approach may contribute to a reduction in the incidence of cardiovascular events among patients with urolithiasis. Future prospective studies should be structured to determine whether interventions targeting kidney stones might mitigate the healthcare costs associated with CVDs[6], thereby potentially decreasing the need for unnecessary diagnostic procedures and pharmacological treatments in patients over the long term”.

5. Additionally, language issues—such as “did not explained” instead of “did not explain” and phrases like “consent of participation”—negatively impact readability. A comprehensive proofreading or professional language review is strongly recommended.

*Reply*: We sincerely thank the reviewer for careful reading. As suggested by the reviewer, we have corrected the “did not explain” into “did not explained”.

We tried our best to improve the manuscript and made some changes to the manuscript. These changes will not influence the content and framework of the paper. And here we did not list the changes but marked in blue in the revised paper. We appreciate for Editors/Reviewers’ warm work earnestly and hope that the correction will meet with approval.

Reference

1.	Current Opinion in Nephrology and Hypertension. Current world literature. Current opinion in nephrology and hypertension. 2010;19(3):315-32. Epub 2010/04/16. doi: 10.1097/MNH.0b013e3283398422. PubMed PMID: 20393288.

2.	Fang H, Deng J, Chen Q, Chen D, Diao P, Peng L, et al. Univariable and multivariable mendelian randomization study revealed the modifiable risk factors of urolithiasis. PloS one. 2023;18(8):e0290389. Epub 2023/08/25. doi: 10.1371/journal.pone.0290389. PubMed PMID: 37624788; PubMed Central PMCID: PMCPMC10456171.

3.	Norman G, Monteiro S, Salama S. Sample size calculations: should the emperor's clothes be off the peg or made to measure? BMJ (Clinical research ed). 2012;345:e5278. Epub 2012/08/25. doi: 10.1136/bmj.e5278. PubMed PMID: 22918496.

4.	Obligado SH, Goldfarb DS. The association of nephrolithiasis with hypertension and obesity: a review. American journal of hypertension. 2008;21(3):257-64. Epub 2008/01/26. doi: 10.1038/ajh.2007.62. PubMed PMID: 18219300.

5.	Taylor EN, Stampfer MJ, Mount DB, Curhan GC. DASH-style diet and 24-hour urine composition. Clinical journal of the American Society of Nephrology : CJASN. 2010;5(12):2315-22. Epub 2010/09/18. doi: 10.2215/cjn.04420510. PubMed PMID: 20847091; PubMed Central PMCID: PMCPMC2994094.

6.	Muschialli L, Mannath A, Moochhala SH, Shroff R, Ferraro PM. Epidemiological and biological associations between cardiovascular disease and kidney stone formation: A systematic review and meta-analysis. Nutrition, metabolism, and cardiovascular diseases : NMCD. 2024;34(3):559-68. Epub 2024/03/03. doi: 10.1016/j.numecd.2023.09.011. PubMed PMID: 38431384.
